# Gallbladder wall abnormality in biliary atresia of mouse *Sox17*^+/−^ neonates and human infants

**DOI:** 10.1242/dmm.042119

**Published:** 2020-04-03

**Authors:** Mami Uemura, Mayumi Higashi, Montri Pattarapanawan, Shohei Takami, Naoki Ichikawa, Hiroki Higashiyama, Taizo Furukawa, Jun Fujishiro, Yuki Fukumura, Takashi Yao, Tatsuro Tajiri, Masami Kanai-Azuma, Yoshiakira Kanai

**Affiliations:** 1 Department of Veterinary Anatomy, the University of Tokyo, Tokyo 113-8657, Japan; 2 Department of Experimental Animal Model for Human Disease, Center for Experimental Animals, Tokyo Medical and Dental University, Tokyo 113-8510, Japan; 3 Department of Pediatric Surgery, Kyoto Prefectural University of Medicine, Kyoto 602-8566, Japan; 4 Department of Pediatric Surgery, the University of Tokyo, Tokyo 113-0033, Japan; 5 Department of Human Pathology, Juntendo University, Tokyo 113-8421, Japan

**Keywords:** SOX17, Cholecystitis, Peribiliary gland, PBG, Pseudopyloric gland, PPG, Human, Mouse, Biliary atresia

## Abstract

Biliary atresia (BA) is characterized by the inflammation and obstruction of the extrahepatic bile ducts (EHBDs) in newborn infants. SOX17 is a master regulator of fetal EHBD formation. In mouse *Sox17*^+/−^ BA models, SOX17 reduction causes cell-autonomous epithelial shedding together with the ectopic appearance of SOX9-positive cystic duct-like epithelia in the gallbladder walls, resulting in BA-like symptoms during the perinatal period. However, the similarities with human BA gallbladders are still unclear. In the present study, we conducted phenotypic analysis of *Sox17*^+/−^ BA neonate mice, in order to compare with the gallbladder wall phenotype of human BA infants. The most characteristic phenotype of the *Sox17*^+/−^ BA gallbladders is the ectopic appearance of SOX9-positive peribiliary glands (PBGs), so-called pseudopyloric glands (PPGs). Next, we examined SOX17/SOX9 expression profiles of human gallbladders in 13 BA infants. Among them, five BA cases showed a loss or drastic reduction of SOX17-positive signals throughout the whole region of gallbladder epithelia (SOX17-low group). Even in the remaining eight gallbladders (SOX17-high group), the epithelial cells near the decidual sites were frequently reduced in the SOX17-positive signal intensity. Most interestingly, the most characteristic phenotype of human BA gallbladders is the increased density of PBG/PPG-like glands in the gallbladder body, especially near the epithelial decidual site, indicating that PBG/PPG formation is a common phenotype between human BA and mouse *Sox17*^+/−^ BA gallbladders. These findings provide the first evidence of the potential contribution of SOX17 reduction and PBG/PPG formation to the early pathogenesis of human BA gallbladders.

This article has an associated First Person interview with the joint first authors of the paper.

## Abstract (precis)

**Summary:** Metaplastic gland formation in gallbladder walls is seen in both human BA and the mouse *Sox17*-haploinsufficient BA model, indicating its contribution to the pathogenesis of human BA.

## INTRODUCTION

Biliary atresia (BA) occurs in one out of every 10,000-15,000 live births, and causes bile duct inflammation because of the blockage of bile flow during the perinatal period (Hartley et al., 2009; Mieli-Vergani and Vergani, 2009). Bile duct injury in extrahepatic bile ducts (EHBDs) may occur in the early pathogenesis of BA (Bezerra et al., 2018), possibly through viral infection (Averbukh and Wu, 2018), toxin exposure (Lorent et al., 2015) and/or individual genetic/epigenetic predisposition (Girard and Panasyuk, 2019) during fetal and perinatal periods (Mack and Sokol, 2005; Nakamura and Tanoue, 2013; Davenport, 2016). BA is traditionally classified into two forms: an ‘embryonic’ form in a minority (30%) of cases and a ‘perinatal’ form in the majority (70%) of patients (Balistreri et al., 1996; Bezerra, 2005; Makin and Davenport, 2017). The ‘embryonic’ form of BA has been described to be caused by the defective formation of the EHBD system during early-to-late organogenic stages, and this form includes the cystic BA and biliary atresia splenic malformation (BASM) syndrome. In contrast, the ‘perinatal’ form mainly consists of ‘isolated’ BA, the largest group with neither clear etiology nor any appreciable defects in other tissues/organs except for the bile ducts (Kelay and Davenport, 2017). Despite these potential heterogeneous causes, one of the most reliable characters of BA is the presence of gallbladder abnormality, such as an echogenic non-identical, atrophic, non-contractile and/or irregularly shaped gallbladder without a definable luminal wall (Tan Kendrick et al., 2003; Kanegawa et al., 2003; Zhou et al., 2016; Aziz et al., 2011; Hwang et al., 2018). However, the pathological phenotypes and their causes in human BA gallbladders are unclear.

Among various animal BA models (Uemura et al., 2013; Waisbourd-Zinman et al., 2016; Higashiyama et al., 2017; Yang et al., 2018; Oetzmann von Sochaczewski, et al., 2018), one model for the ‘embryonic’ BA form is the haploinsufficient BA mouse of the SRY-related HMG box factor-17 (*Sox17*) gene, a master regulator for EHBDs in various vertebrate species including mice and humans (Spence et al., 2009; Uemura et al., 2010, 2015). The SOX17-positive EHBD progenitors within the ventral foregut region (Uemura et al., 2010, 2015) proliferate and expand distally far from the duodenum. This leads to the formation of one long and narrow tube of fetal gallbladder and cystic duct, accompanied by the maintenance of high SOX17 expression in the gallbladder domain during organogenic stages (Uemura et al., 2013). In *Sox17*-heterozygous (*Sox17*^+/−^) mouse embryos, reduced SOX17 expression induces hypoplastic gallbladder by the late-organogenic stages. The defective proliferation and shedding of the *Sox17^+/−^* gallbladder epithelia, along with their transdifferentiation into SOX9-positive cystic duct-like epithelia, causes the onset of inflammation and obstruction in downstream EHBDs (i.e. cystic duct, hepatic duct and common bile duct) in most *Sox17*^+/−^ neonates (Uemura et al., 2013; Higashiyama et al., 2017). In the toxin-mediated BA model, exposure to a plant toxin, biliatresone, also led to reduced SOX17 expression in an EHBD spheroid culture, resulting in the loss of epithelial polarity and luminal obstruction, which are similar to BA symptoms (Waisbourd-Zinman et al., 2016). Therefore, it is likely that insufficient SOX17 levels in fetal EHBDs may mediate bile duct injury in BA-like pathogenesis in these two animal models.

SOX17-positive gallbladder progenitors contribute to the great majority of the fetal EHBD system before the first biliary excretion into the fetal duodenum (Spence et al., 2009; Uemura et al., 2010). Therefore, it is possible that defects and/or damage in the epithelial barrier occur in the gallbladder walls at the fetal stage in human BA cases (Davenport, 2016; Verkade et al., 2016). Moreover, such bile duct injuries may be repaired by EHBD progenitors in peribiliary glands (PBGs), so-called pseudopyloric glands (PPGs) in pathology (Cardinale et al., 2012; Carpino et al., 2012; DiPaola et al., 2013), which are alveolar-like glands composed of serous and mucinous acini (Hopwood et al., 1988; Sugiura and Nakanuma, 1989; Zimmermann, 2016). In both humans and mice in the normal healthy state, few glandular structures exist in the gallbladder, but SOX9-positive PBGs are distributed widely throughout the ductular walls of cystic ducts and other proximal parts of the biliary tract, indicating a possible role in the physiological maintenance of the barrier function of bile duct epithelia (Furuyama et al., 2011; de Jong et al., 2018). However, the SOX17/SOX9 expression profiles and PBG/PPG dynamics in human BA infants, despite having a well-preserved gallbladder in some of the ‘isolated’ BA cases, remain to be characterized.

In the present study, we examined the histopathological phenotypes of gallbladder abnormalities in human BA and mouse *Sox17*^+/−^ pups, focusing on the SOX17 expression and PBG/PPG structures in the gallbladder walls.

## RESULTS

### Increased density of PBG/PPG-like glands in the gallbladder wall of *Sox17*^+/−^ neonates

**Fig. 1.**
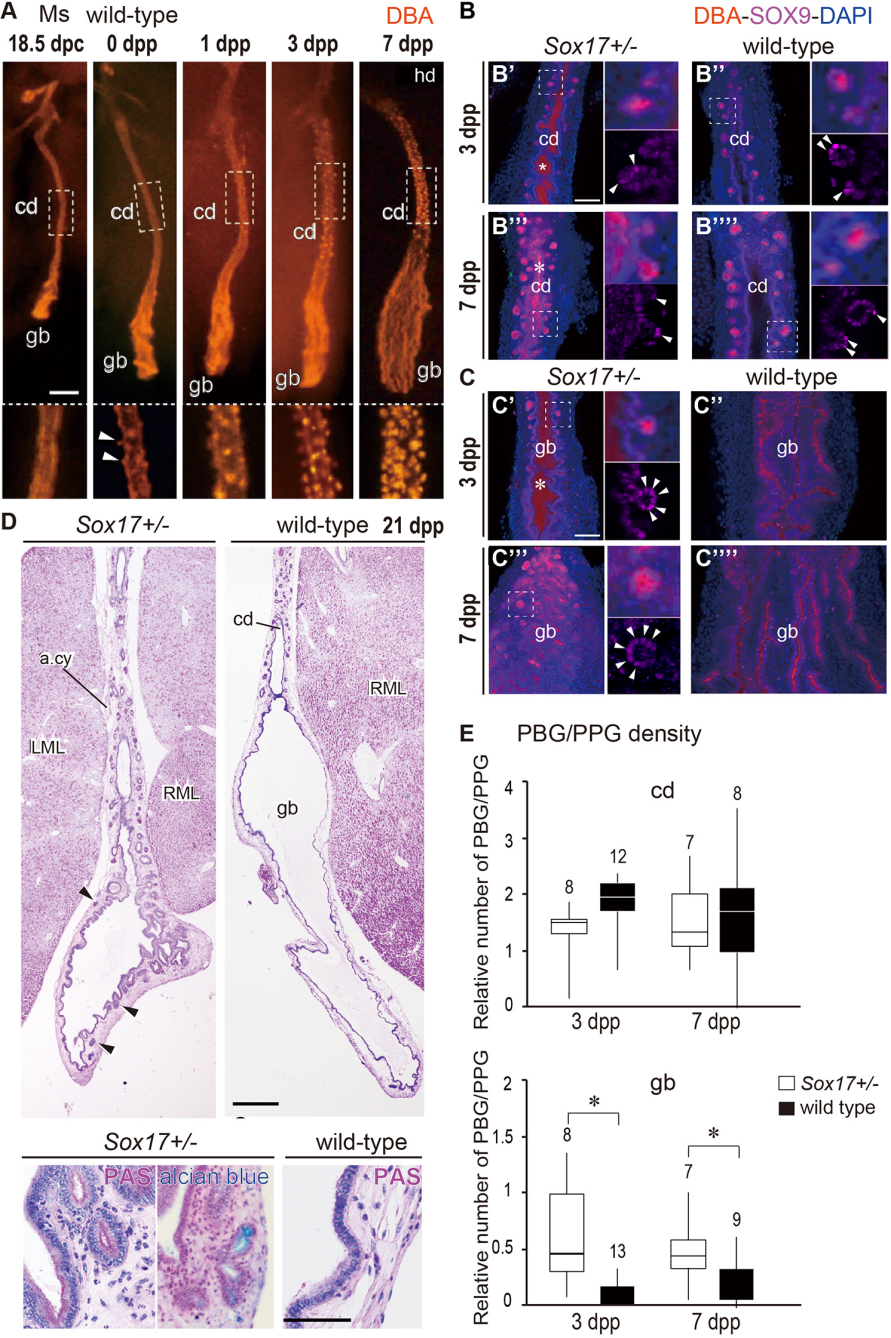
**Increased density of PBG-like glands in mouse *Sox17*^+/−^ gallbladders.** (A) DBA-stained EHBDs of wild-type neonates (from 18.5 dpc to 7 dpp). The lower panels display higher magnification views of the cystic duct (boxed region) in the upper panels. The arrowheads indicate the first sign of PBG formation in the DBA-positive cystic duct wall at 0 dpp. (B,C) Confocal microscope images of whole-mount DBA/SOX9 double-stained EHBD samples of cystic duct (B) and gallbladder (C) from *Sox17*^+/−^ (left) and wild-type (right) mice at 3 and 7 dpp. Dotted white boxes indicate areas shown at higher magnification in the upper right insets; lower right insets show the SOX9 expression patterns in these regions. Arrowheads indicate SOX9-positive cells. Asterisks in the luminal space of the *Sox17*^+/−^ bile ducts (B′,B‴,C′) indicate the aberrant accumulation of DBA-positive cell debris. (D) H&E, PAS and Alcian blue staining of the sagittal EHBD sections including gallbladder (gb) and cystic duct (cd) in mouse *Sox17*^+/−^ and wild-type littermates at 21 dpp. The PBG-like glands (also known as PPG) are indicated by arrowheads. Higher magnification is shown in the lower insets. (E) Morphometric analyses using whole-mount DBA-stained samples (box plots), showing PBG/PPG count in the cystic duct (upper) and gallbladder (lower) of *Sox17*^+/−^ (white bar) and wild-type (black bar) littermates. PBG/PPG density in the gallbladder was significantly higher in *Sox17*^+/−^ pups than in wild-type littermates. The upper number in each box is the sample number. The horizontal line within each box is the median value (50th percentile), box indicates first to third interquartile ranges and whiskers indicate the highest/lowest values. Each PBG/PPG density in *Sox17*^+/−^ pups versus that in wild-type pups (mean±s.e.m.) are as follows: 1.4±0.2 versus 1.9±0.1 at 3 dpp, 1.6±0.3 versus 1.6±0.4 at 7 dpp in the cystic duct region; 0.6±0.2 versus 0.1±0.0 at 3 dpp, 0.5±0.1 versus 0.2±0.1 at 7 dpp in the gallbladder region. *P<0.05, Student's two-tailed *t*-test. a.cy, cystic artery; cd, cystic duct; gb, gallbladder; LML, left medial lobe of liver; Ms, mouse; hd, hepatic duct; RML, right medial lobe of liver. Scale bars: 100 µm.

In the *Sox17*^+/−^ mouse BA model, the ectopic appearance of cystic duct-like epithelia in the gallbladder domain occurs during the fetal stages before the first secretion of bile fluid from the fetal liver [by 15.5 days post-coitum (dpc); Uemura et al., 2013; Higashiyama et al., 2017]. One prominent histological character of the cystic duct walls is the PBGs, which are formed in EHBDs but not the gallbladder (see review by de Jong et al., 2018). First, we observed the normal development patterns of PBGs in the gallbladder and cystic duct regions of wild-type mouse neonates (Fig. 1). Whole-mount *Dolichos biflorus* agglutinin (DBA) staining visualized the epithelial architecture of the gallbladder and cystic duct of the EHBDs isolated from 18.5 dpc to 7 days post-partum (dpp) (Fig. 1A-C). In the cystic duct region, as well as in the gallbladder, PBG structures were not identified in the wild-type embryos before birth. PBG-like bud structures first arose in the cystic duct region at 0 dpp (arrowheads in lower plates of Fig. 1A). Subsequently, the complete PBGs were rapidly formed in the cystic duct region from 1 to 7 dpp (Fig. 1A; right plates in Fig. 1B). In contrast, long and narrow epithelial folds were formed along the proximal-to-distal axis of the developing gallbladder (upper plates in Fig. 1A; right plates in Fig. 1C), and PBG-like structures could be rarely found in the wild-type gallbladder at 1-7 dpp.

Next, we examined PBG formation in the *Sox17*^+/−^ pups at 3 and 7 dpp (left plates in Fig. 1B,C). DBA/SOX9 double whole-mount immunostaining revealed that DBA-positive materials (i.e. cellular debris) were frequently observed within the luminal space of both gallbladder and cystic duct in *Sox17*^+/−^ neonates, but not in the healthy wild-type littermates (asterisks in Fig. 1B,C), indicating inflammation and scarring of fetal cholecystitis in *Sox17*^+/−^ neonates (Higashiyama et al., 2017). Even under this condition, SOX9/DBA-positive PBGs-like glands (PPGs) were properly formed in the cystic duct of the *Sox17*^+/−^ pups, as well as in the wild-type pups (Fig. 1B). In contrast, in the *Sox17*^+/−^ gallbladders, SOX9-positive PBG/PPG structures were frequently found in the gallbladder region (left plates in Fig. 1C). This is in sharp contrast to the epithelial folds without the bud-like structure observed in the wild-type littermates (right plates in Fig. 1C). In the post-weaning *Sox17*^+/−^ mice at 3 weeks old, we confirmed the broad distribution of Alcian blue-stained PBG/PPG structures along the gallbladder wall (arrowheads in left plates of Fig. 1D), but observed few glands in the gallbladders of wild-type littermates (right plates in Fig. 1D). Quantitative analysis using whole-mount-stained samples confirmed a significant increase in PBG/PPG density in the gallbladder, but not in the cystic duct, in *Sox17*^+/−^ pups compared with wild-type littermates at 3 and 7 dpp (Fig. 1E). These findings indicate that such a frequent appearance of PBG/PPG structures is the most prominent character in the gallbladders of the *Sox17*^+/−^ neonates, which is consistent with the embryonic cholecystitis, together with ectopic appearance of SOX9-positive cystic duct-like epithelia, in the *Sox17*^+/−^ gallbladders at late-organogenic stages (Higashiyama et al., 2017).

### Classification of SOX17-high and -low groups in human BA gallbladders

**Fig. 2.**
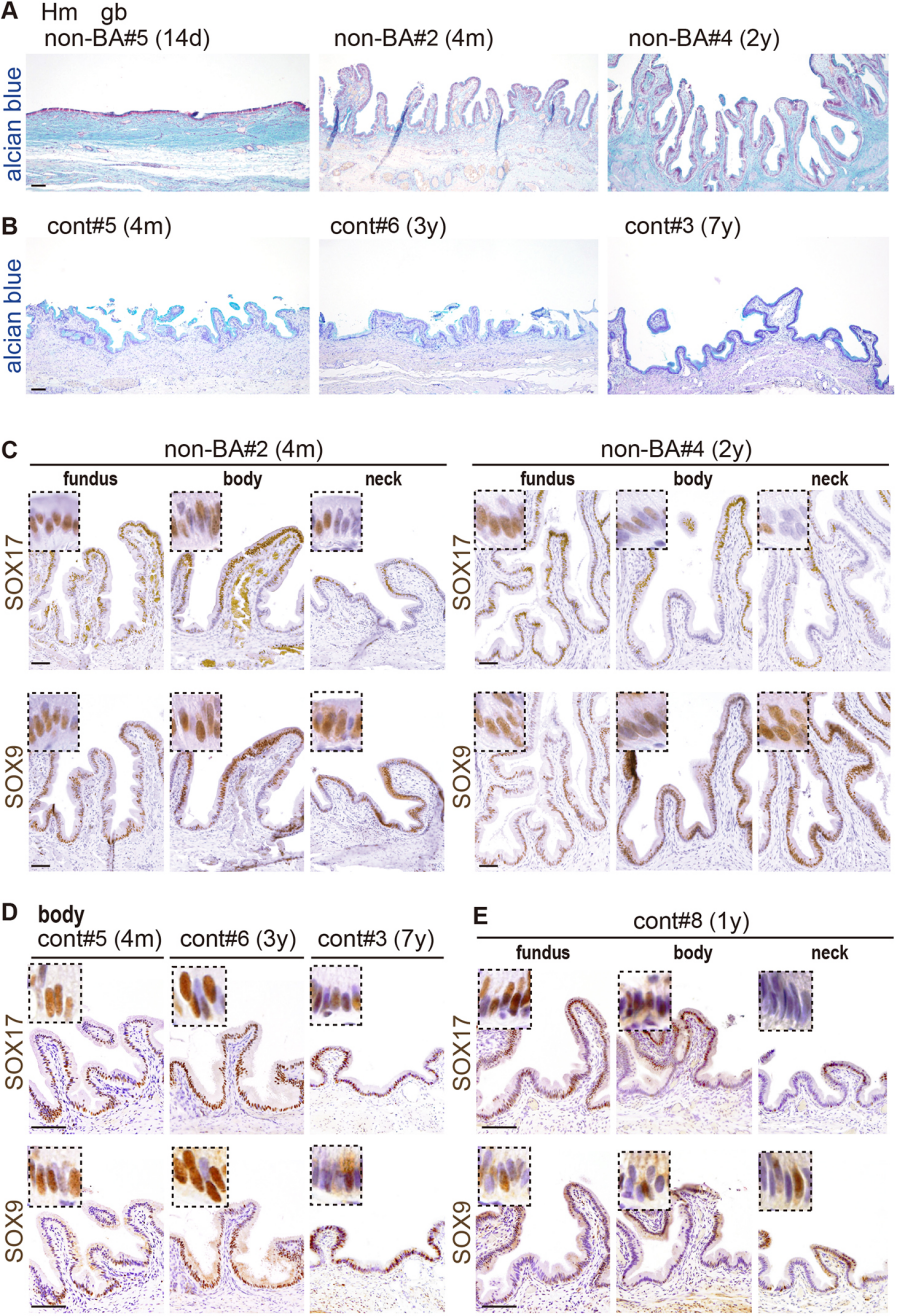
**The expression profiles of SOX17 and SOX9 in human non-BA and control (cont) gallbladder walls.** (A,B) Lower-magnification images (Alcian blue staining) of the gallbladder walls (gallbladder body) in non-BA and control patients (14-day∼7-year old). (C-E) Anti-SOX17 (upper) and SOX9 (lower) immunostaining of two serial sections of the fundus, body and neck regions of the gallbladders in human non-BA (C) and control (D,E) infants, in addition to the SOX17 and SOX9 expression profiles in the gallbladder body of three control patients at 4 months, 3 years and 7 years old (D). Insets show higher-magnification images of SOX17/SOX9-positive epithelial cells. Hm, human. Scale bars: 100 µm.

For pathological analysis of human gallbladders, we first examined the SOX17/SOX9 expression profiles in seven non-BA [six congenital biliary dilation (CBD) cases, one gallstone (GS) case] and eight control [one pancreatoblastoma (PB) case, seven hepatoblastoma (HB) cases] gallbladders (Fig. 2; Table 1). In human non-BA gallbladders, epithelial folds became evident by 4 months old in both non-BA and control specimens (Fig. 2A,B; Table 1), suggesting a similar developmental profile to mouse gallbladders at 3-7 days old (Fig. 1A,C). The epithelial fold structures were stably maintained in the control gallbladders in the range of 4 months to 9 years old (Fig. 2B; Table 1), but some non-BA gallbladders (e.g. non-BA#4) displayed epithelial hyperplasia owing to the chronic inflammation (i.e. bile retention/pancreatic juice reflux) at 2 years old (right plate in Fig. 2A). In both non-BA and control gallbladders (a range of 14 days to 9 years of age), SOX9-positive epithelial cells were widely distributed throughout the entire gallbladder, including the fundus, body and neck regions (Fig. 2C-E; also see Table 1). In the same gallbladder specimens, SOX17-positive epithelial cells were also detectable throughout the entire region (Fig. 2C-E), although reduced SOX17 expression was observed in the proximal neck region (‘neck’ in Fig. 2C,E). Such human SOX17 expression profiles are similar to those observed in mouse gallbladder, exhibiting higher SOX17 expression in the distal gallbladder region than in the proximal neck in fetal stages (Uemura et al., 2013; Higashiyama et al., 2017).

**Table 1.**
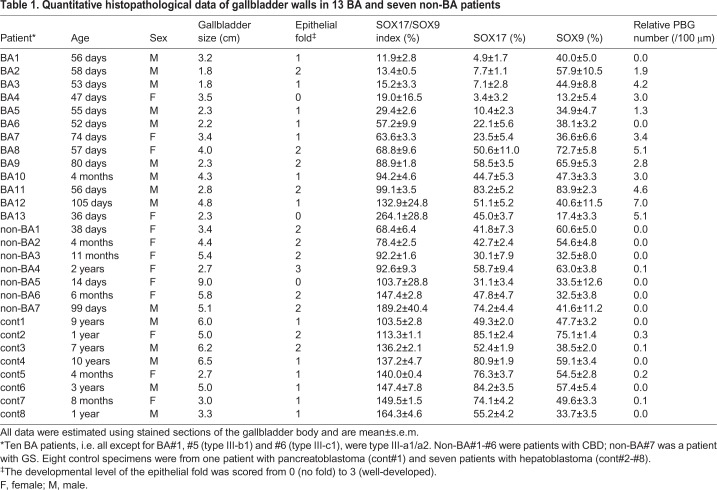
**Quantitative histopathological data of gallbladder walls in 13 BA and seven non-BA patients**

**Fig. 3.**
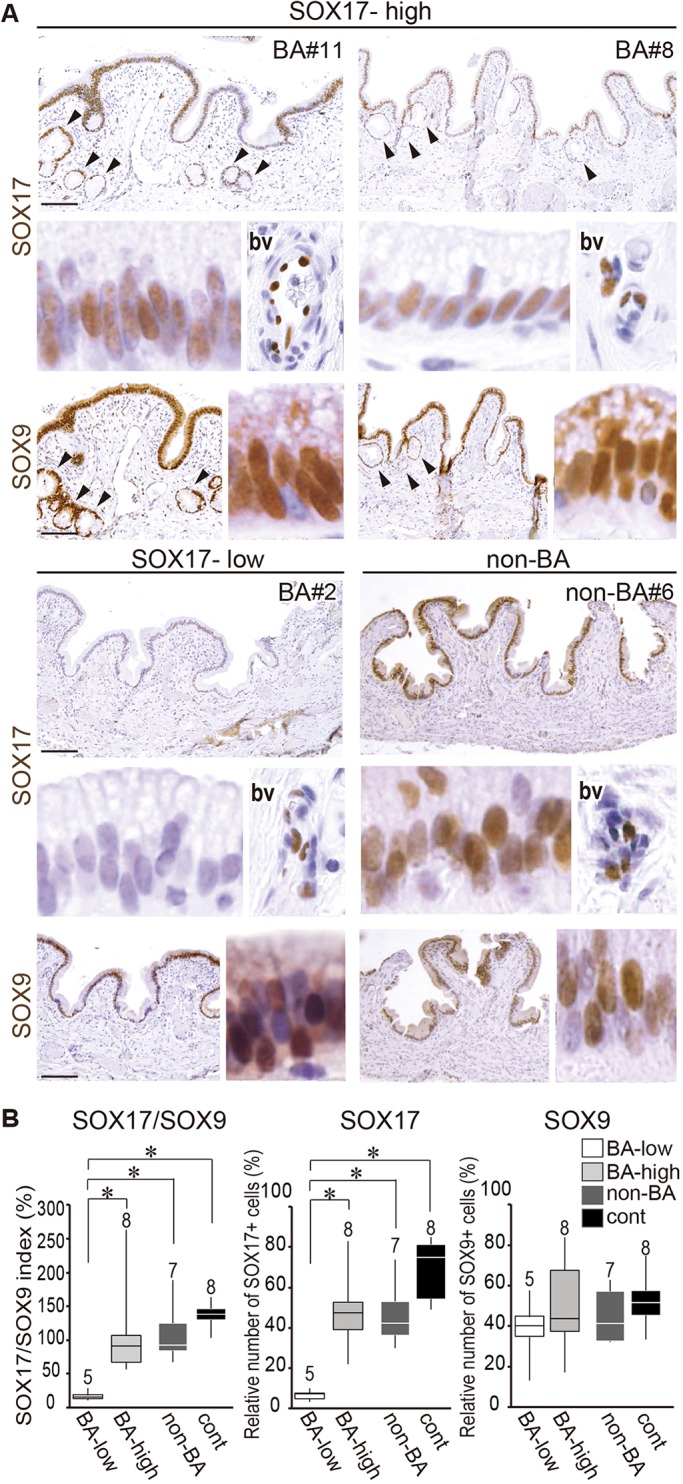
**The expression profiles of SOX17 and SOX9 in human BA gallbladder walls.** (A) Anti-SOX17 (upper) and anti-SOX9 (lower) immunostaining of two serial sections of three BA gallbladders [gallbladder body; two-SOX17-high (BA-high) and one SOX17-low (BA-low)]. Insets show higher-magnification images of the gallbladder epithelia in each sample. A non-BA sample (non-BA#6) is also shown. Insets labeled ‘bv’ display the SOX17-positive endothelial cells observed in the same stained section. Arrowheads show PBG/PPG-like glands. (B) Morphometric analyses using the SOX17/SOX9-stained sections (box plots), displaying the SOX17/SOX9 indices (left) and relative numbers of SOX17-positive (center) or SOX9-positive (right) epithelial cells in the gallbladder body of each group. The horizontal line within each box is the median value, box indicates first to third interquartile ranges and whiskers indicate the highest/lowest values. *P<0.05, Student's two-tailed unpaired *t*-test. bv, blood vessel; cont, control. Scale bars: 100 µm.

Next, we examined the 13 BA gallbladders that exhibited persistent epithelial structures out of 29 BA cases. Serial sections of the gallbladder body were examined using anti-SOX17/SOX9 immunohistochemistry, then classified into the two groups (SOX17-high and -low) based on the relative number of SOX17-positive epithelial cells in the gallbladder body, as shown in Fig. 3 and Table 1. In brief, in the SOX17-high group (BA#6-13) an average of 47.3±6.9% (mean±s.e.m.) of epithelial cells were positive for anti-SOX17 by immunostaining in each section (upper plates in Fig. 3A). The proportion of SOX17-positive cells was similar to that in the non-BA group (non-BA; average 46.6±5.9%), but less than that of the control group (cont; average 69.7±5.3%) (Fig. 2C-E and Fig. 3A; middle graph in Fig. 3B). In the SOX17-low group (BA#1-5), the proportion of SOX17-positive cells was significantly lower (6.7±1.2%) compared with the other three groups of the SOX17-high BA, non-BA and control groups (SOX17-low; Fig. 3A; middle graph in Fig. 3B). In contrast, no appreciable differences were seen in the proportion of SOX9-positive cells detectable among the SOX17-high BA, SOX17-low BA, non-BA and control groups (right graph in Fig. 3B).

As shown in Fig. 3B (left graph) and Tables 1 and 2, we also estimated the SOX17/SOX9 index [i.e. a ratio of SOX17-positive epithelial cells to SOX9-positive cells (×10^−2^)] as the ectopic appearance index of the SOX9-positive cystic duct-like epithelial cells in fetal gallbladders of the *Sox17*^+/−^ and wild-type littermates (Higashiyama et al., 2017). In the SOX17-low BA group, the average SOX17/SOX9 index was 17.8±3.1% (range: 11.9-29.4%) in the gallbladder body region. The average SOX17/SOX9 index in the SOX17-high BA group was significantly higher at 108.6±23.8% (range: 57.2-264.1%), which was similar to the non-BA (110.3±16.2%; range: 68.4-189.2%) and control (136.2±6.9%; range: 103.5-164.0%) groups. These data indicate that 5 of 13 human BA gallbladders exhibited a significantly reduced number of SOX17-positive epithelial cells in their walls.

**Table 2.**
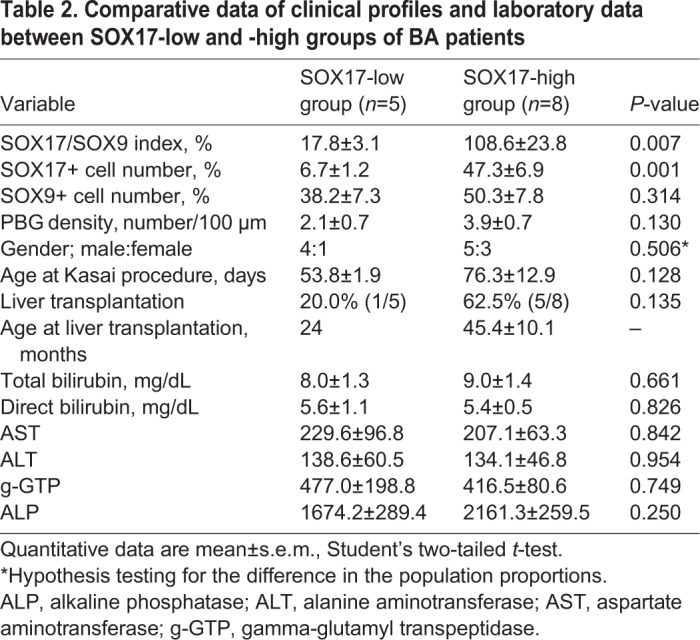
**Comparative data of clinical profiles and laboratory data between SOX17-low and -high groups of BA patients**

Analyzing patient clinical information showed that patients in the SOX17-low group were slightly (non-significantly) younger and more likely to have had early Kasai operations than those in the SOX17-high group (Table 2). Based on clinical serum data, we did not find any correlation between conventional biochemical indices of liver damage [aspartate aminotransferase (AST), alanine aminotransferase (ALT), alkaline phosphatase (ALP)] and bile duct obstruction [total and direct bilirubin, and gamma-glutamyl transpeptidase (g-GTP)] in the two BA groups (Table 2).

### Appearance of PBG/PPG structures, especially near the shedding epithelial sites, in human BA gallbladders

**Fig. 4.**
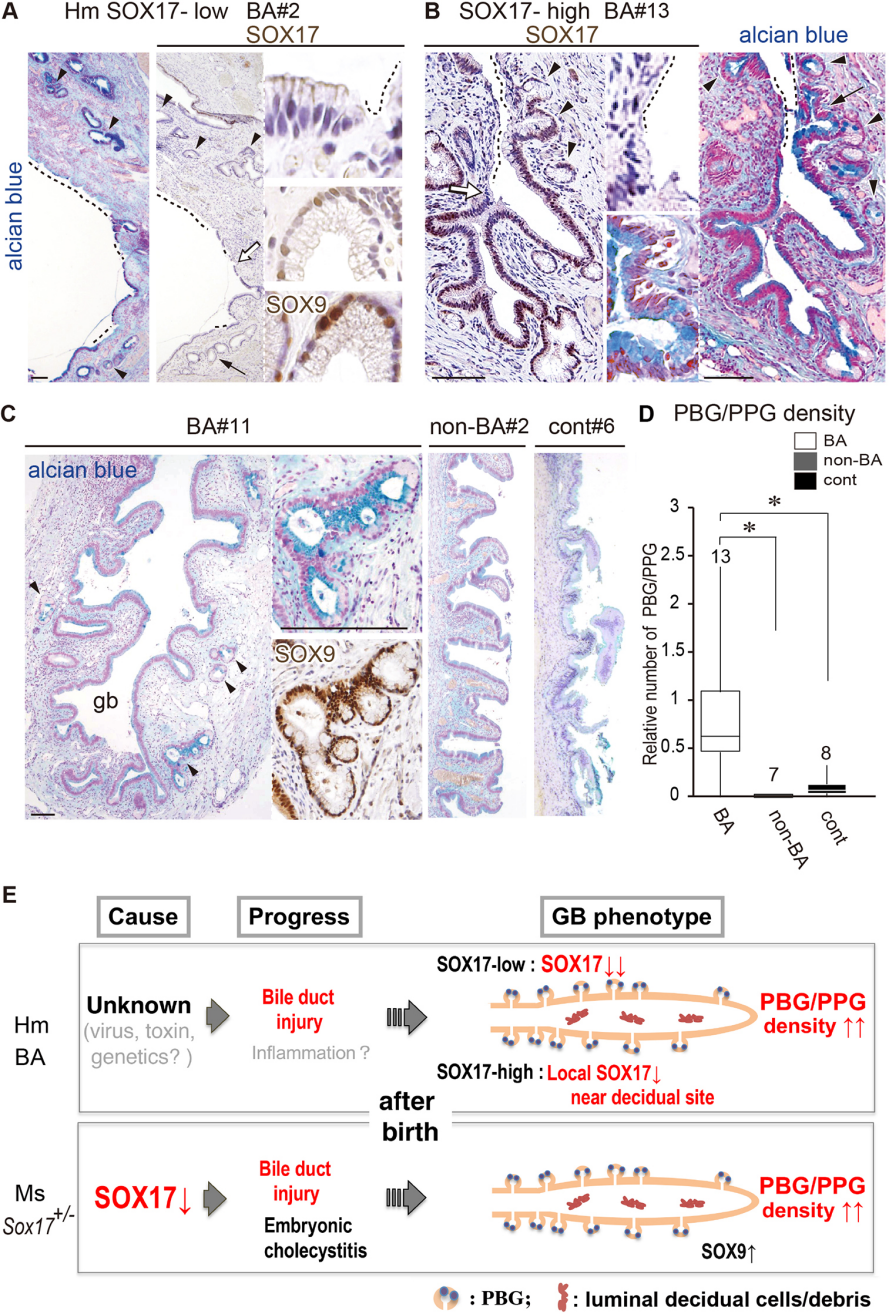
**Appearance of PBG/PPG structures, especially near the decidual sites, in human BA gallbladders.** (A,B) Alcian blue and anti-SOX17 staining of gallbladders from SOX17-low (BA#2; A) and SOX17-high (BA#13; B) groups, showing the epithelial decidual sites (dashed lines) and PBG/PPG-like glands (arrowheads) of human BA gallbladders. Insets show higher-magnification images of the SOX17-negative epithelial site or glands, indicated by a white or black arrow, respectively. In the lower inset of A, the SOX9-stained image of the same gland is also shown. (C) Alcian blue and anti-SOX9 staining, showing PBG/PPG-like glands (arrowheads) in the gallbladder walls (BA#11 in SOX17-high). Images of non-BA (non-BA#2) and control (cont#6) gallbladder walls are also shown. (D) Morphometric analyses (box plots) showing a significant increase in the abundance of PBG/PPG-like glandular structures (per 100 µm length) in BA compared with non-BA and control gallbladders. The horizontal line within each box is the median value, box indicates first to third interquartile ranges and whiskers indicate the highest/lowest values. *P<0.05, Student's two-tailed, unpaired *t*-test. (E) Schematic showing the gallbladder wall phenotypes shared between human BA patients and mouse *Sox17*^+/−^ neonates (red) (Uemura et al., 2013; Higashiyama et al., 2017). The potential causes and progression of human gallbladder pathogenesis (Bezerra et al., 2018) are also indicated (gray). gb, gallbladder; Hm, human; Ms, mouse. Scale bars: 100 µm.

In all human BA gallbladders, epithelial deciduation was frequently found in both the SOX17-low and SOX17-high groups (dashed lines in Fig. 4A,B), although there may have been some artificial gallbladder wall damage in some human control patients, possibly because of the delay of formalin fixation (right plate in Fig. 4C). Even in the SOX17-high group, certain epithelial cells near the shredding epithelia region showed negative or weak anti-SOX17 staining intensity, suggesting a possible contribution of reduced SOX17 expression during epithelial deciduation in human BA. This histopathological analysis revealed that the appearance of PBG/PPG-like glands was frequently observed in both the SOX17-low group and the SOX17-high group (arrowheads in Fig. 4A-C), especially near the shedding epithelia region. This is in sharp contrast to non-BA and control human gallbladders, in which few glandular structures were observed beneath the surface epithelia (Fig. 4C). Morphometric analysis also confirmed a significant increase in PBG/PPG density in human BA infants compared with non-BA and control human cases (Fig. 4D; also see right-most column in Table 1). These data suggest that the PBG/PPG formation in the gallbladder is a common pathological character between human BA infants and mouse *Sox17*^+/−^ neonates.

The gallbladder phenotypes, including their potential etiology and progression in human BA infants and mouse *Sox17*^+/−^ neonates, are summarized in Fig. 4E.

## DISCUSSION

The present analyses showed that the spatiotemporal SOX17-expression profiles in human control/non-BA gallbladders are similar to those in mouse gallbladders during postnatal development. In both human and mouse gallbladders, SOX17 expression appears to be higher in the distal (fundus and body) region than the proximal (neck) region, although SOX17 expression in fetal mouse gallbladders decreases throughout perinatal development (Fig. 2; Fig. S1; Uemura et al., 2013). Whole-mount DBA staining of mouse EHBDs demonstrated that PBG-like glands formed in the cystic duct region of mouse neonates soon after birth. These PBGs first appeared at the newborn stage and rapidly developed within several days after birth (Fig. 1A). This developmental process is also similar to the PBGs in human EHBDs, which are very sparse in the fetal period (mean gestational age 4 months) and much more abundant in newborn babies (mean gestational age 9.25 months) (Spitz and Petropoulos, 1979). Given the extensive anatomical similarity between mice and humans in the extrahepatic biliary tracts and the associated blood vessels, nerves and smooth muscles (Higashiyama et al., 2016), the *Sox17^+/−^* mouse gallbladder is a useful model for human gallbladder pathogenesis caused by bile duct injury via decreased barrier function at fetal stages.

Among 13 BA gallbladders, five exhibited reduced numbers of SOX17-positive epithelia in gallbladder walls compared with the remaining eight (SOX17/SOX9 indices: 17.8±3.1% in the SOX17-low group versus 108.6±23.8% in the SOX17-high group). In late stages of mouse EHBD regionalization, persistent SOX17 expression is involved in the specification of the gallbladder domain, whereas SOX9 is also involved in the cystic, hepatic and common bile duct regions, as well as the intrahepatic ducts (Spence et al., 2009; Uemura et al., 2010; Poncy et al., 2015). SOX17-heterozygotic EHBD epithelia lead to cell autonomous shedding in the gallbladder domain, which is accompanied by higher *Cxcl10* expression, not only *in vivo* but also under explant culture conditions using isolated gallbladder primordia (Higashiyama et al., 2017). Moreover, in another BA model using the plant toxin biliatresone, toxin treatment caused extrahepatic cholangiocyte damage and fibrosis through decreased SOX17 expression in three-dimensional spheroid culture of mouse bile duct cells and neonatal extrahepatic duct cells (Waisbourd-Zinman et al., 2016). The present study demonstrated that reduced SOX17 expression is frequently observed in the region near the decidual site, even in SOX17-high human BA cases, in addition to the low SOX17 levels observed in the five SOX17-low BA gallbladders. These findings, therefore, suggest a possible contribution of reduced SOX17 expression levels to the early pathogenesis of some human BA gallbladders.

The most remarkable finding in the present study is that the density of PBG/PPG-like glands are significantly increased in gallbladders in both human BA cases and the mouse *Sox17*^+/−^ BA model, suggesting common characteristics of BA gallbladder pathogenesis between these two species. This is consistent with several previous studies showing the malformation of PBG/PPG-like glands in some severe bile diseases, including polycystic disease, in cases of cirrhosis and cystic/papillary neoplasm (Kida et al., 1992; Bhathal et al., 1996; Hughes et al., 2010; Miyata et al., 2016; Goossens et al., 2017). At present, there are two putative mechanisms for the frequent appearance of glandular structures in BA gallbladders. One is the positive response against bile duct injury owing to the protection and recovery of the epithelial barrier. The PBGs/PPGs secrete mucus and immunoglobulin, thus contributing to the protection of the apical surface of the bile duct epithelia from bile salt toxicity (DiPaola et al., 2013; Hopwood et al., 1988; Sugiura and Nakanuma, 1989). PBGs also contain epithelial cells expressing several biliary and hepatic stem cell markers (including *Sox9*, *Sox17*, *Pdx1* and *Lgr5*), and it has therefore been suggested that they may be the reservoir of epithelial stem/progenitor cells in the biliary tract (Furuyama et al., 2011; Cardinale et al., 2012; Carpino et al., 2012; DiPaola et al., 2013; Lanzoni et al., 2016; Matsui et al., 2018; de Jong et al., 2018, 2019). Therefore, it is possible that the metaplastic PBG/PPG-like glands may serve as a positive response to regenerate the epithelial structure in the BA gallbladders following stress/inflammation during late fetal and perinatal periods.

Another possible mechanism is the ectopic appearance of cystic duct-like epithelia in human BA gallbladders. In *Sox17*^+/−^ gallbladders at a late organogenic stage, transcriptomic analyses revealed the appearance of ectopic cystic duct-like epithelia that are similar to the hepatic and common bile ducts (Higashiyama et al., 2017). In these *Sox17*^+/−^ gallbladders, reduced SOX17 expression induces SOX9-positive characters in gallbladder epithelia, along with reduced proliferation and luminal deciduation (Uemura et al., 2013; Higashiyama et al., 2017). This leads to hypoplastic and non-contractile gallbladders that are similar to the abnormal gallbladders in human BA patients (Tan Kendrick et al., 2003; Kanegawa et al., 2003; Zhou et al., 2016; Aziz et al., 2011; Hwang et al., 2018). Along with the increased PBG/PPG density in *Sox17*^+/−^ gallbladders (Fig. 1), these glandular structures in BA patients may be associated with SOX17 expression levels in gallbladder walls at the fetal stage, at least in some human BA cases in the SOX17-low BA group.

In this study, we mainly used the gallbladders of CBD as non-BA samples. At the time of the operation, the CBD gallbladders showed the pathological characteristics of the ‘chronic’ inflammation in their luminal walls (Kamisawa et al., 2017), despite being at the ‘acute’ inflammation state with the epithelial deciduation of extrahepatic ducts in most of BA patients (Kahn, 2004). The present study showed a slight, albeit not significant, reduction of SOX17-positive cell density even in non-BA gallbladders compared with the control BA ones (Fig. 3B). This is possibly because of the contribution of the inflammation states that affect the SOX17 expression level in both BA and non-BA gallbladders. In contrast, ectopic PBG/PPG appearance in the gallbladder walls was evident in the BA, but not in the CBD cases (Fig. 4C), which may possibly reflect the epithelial damage at the ‘acute’ inflammation phase, rather than at the ‘chronic’ state, in the BA gallbladders.

Finally, among 29 BA gallbladders we identified 13 that exhibited persistent epithelial structures (mostly type-IIIa1/2, but also two III-b1 cases and one III-c1 case). We analyzed SOX17/SOX9 indices and PBG dynamics in these 13 gallbladders, but these parameters remained unclear in the remaining 16 gallbladders (nine type-III-b1, two III-c1/c2 and five III-d cases), which mostly lacked epithelial structures at the time of the Kasai operation. In the Japanese Society of Pediatric Surgeons (JSPS) classification (Kasai et al., 1976; Sinha et al. 2008), type III is the commonest type (∼90%) with the most proximal level of obstruction in the porta hepatis. This is also subclassified into four subtypes depending on the state of the base of the common bile duct (‘a’, patent; ‘b’, fibrous; ‘c’, aplasia; ‘d’, miscellaneous), together with the subtypes at the base of hepatic duct states in the porta hepatis (‘1’, patent/atretic; ‘2’, aplasia). All of the type-a cases sustained the epithelial architecture of the gallbladder walls, in contrast to its severe lack in the other types b-d. As the type-a gallbladders have the luminal connection to the duodenum via the patent common bile duct, such persistent epithelial structures in the selected type-a samples may be possibly associated with the proper drainage of the gallbladder sludge in these patients.

Moreover, we examined 13 BA gallbladders and identified increased PBG/PPG density in 11 specimens including 10 type-a and one type-b cases (Table 1). Among these 11 specimens, ectopic PBG/PPG-like glands were evident (>1.9 per 100 µm) in all of the 10 type-a cases, in contrast to the lowest density (i.e. 1.3 per 100 µm) in one type-b case (BA #5 in Table 1). In contrast, ectopic PBG/PPG-like glands were not detectable in two BA cases of type-b and type-c (BA#1, #6 in Table 1). These two BA patients (BA#1, #6) exhibited severely damaged gallbladder epithelia with advanced liver cirrhosis compared with the other eleven BA cases (data not shown), suggesting the end-stage inflammation before the complete loss of the epithelial structures in these two gallbladders. With regard to such reduced gland densities in the cases with severe gallbladder wall phenotypes, it is possible that, after birth, the ectopic PBG/PPG structures first develop at the acute phase of the inflammation and then become depleted before the complete disappearance of the luminal epithelial structures in some BA gallbladders. Further large-scale histopathological analysis of human BA gallbladders is required to understand the concrete role of the PBG/PPG-like glands in the early pathogenesis of BA gallbladders.

## MATERIALS AND METHODS

### Mouse BA model of *Sox17*^+/−^ neonates

*Sox17*^+/−^ embryos and pups were obtained from wild-type females [C57BL6 (B6) strain; Clea Japan] mated with *Sox17*^+/−^ male mice (Kanai-Azuma et al., 2002) that are intercrossed and maintained at N10-N11 backcross generation to the B6 strain (Uemura et al., 2013). All animal experiments were performed in strict accordance with the Guidelines for Animal Use and Experimentation of the University of Tokyo. All procedures were approved by the Institutional Animal Care and Use Committee of the Graduate School of Agricultural and Life Sciences at the University of Tokyo (approval ID: P13-763).

### Human BA patients

Among 29 BA samples from 2006 to 2017 held at the University Hospital of Kyoto Prefectural University of Medicine, 13 BA cases (eight type III-a1, two type III-a2, two III-b1 and one III-c1 cases; all of them belonged to the ‘isolated’ BA category) were selected based on histological examination of the remnant gallbladder walls resected at Kasai portoenterostomy. These 13 gallbladder walls sustained the epithelial architecture of the gallbladder, in contrast with its almost complete lack in the remaining 16 BA gallbladders (nine type-III-b1, two III-c1/c2 and five III-d cases). The type and subtypes of each BA patient were defined according to JSPS classification (Kasai et al., 1976; Sinha et al. 2008). Clinical data for BA infants were obtained retrospectively from clinical files (Tables 1 and 2). The Ethics Committee of the Kyoto Prefectural University of Medicine approved all studies.

Seven non-BA infants, including six with CBD and one with a GS held at Kyoto Prefectural University of Medicine, comprised the non-BA group. Moreover, we used eight normal gallbladder specimens from seven infants with HB and one with PB held at the hospitals of Juntendo University and the University of Tokyo. All human samples in this study were collected and analyzed in strict accordance with the Guidelines of the Kyoto Prefectural University of Medicine, Juntendo University, and the University of Tokyo.

### Sampling of gallbladders

Mouse tissue samples were fixed in 4% paraformaldehyde (PFA) in phosphate-buffered saline (PBS) for 12 h at 4°C. They were then dehydrated in ethanol, replaced by xylene and embedded in paraffin. For whole-mount experiments, some tissue samples were subsequently washed with PBS with 0.05% Tween 20 (PBST) and stored in 70% methanol at −20°C. Human gallbladder specimens at Kasai portoenterostomy were immediately fixed in 10% formalin solution. Each gallbladder was cut into small segments (∼3-4 mm in length) along the distal-to-proximal axis (from the fundus to the neck region), and then dehydrated and embedded in paraffin. The serial deparaffinized sections (4 µm in thickness) of the fundus, body and neck segments of each gallbladder were used for subsequent histopathological analyses, as described below.

### Histology and immunohistochemistry

For human and mouse tissue samples, all sections were subjected to conventional hematoxylin and eosin (H&E), periodic acid Schiff (PAS) and Alcian blue staining.

For immunohistochemistry, deparaffinized sections were incubated with goat anti-SOX17 (1/100; R&D Systems, AF1924) or rabbit anti-SOX9 (1/1000; Millipore, AB5535) at 4° overnight, then washed with PBS. The reactions were visualized with biotin-conjugated secondary antibodies (1/400; Vector Laboratories, BA5000 and BA1000) in combination with ABC kits (Vector Laboratories). The SOX17-positive signal intensity in each section was estimated at standard signal intensity in the SOX17-positive endothelial cells of the small vessels within the same sections (see ‘bv’ in Fig. 2C).

For the whole-mount immunohistochemistry analysis of mouse EHBDs, the specimens were incubated with primary antibody (rabbit anti-SOX9; 1/500) for 2 days at room temperature, washed with PBST for 1 day, and then incubated with secondary antibodies conjugated with Alexa-594 [diluted 1/500 in blocking reagent (TNB); PerkinElmer, FP1020] for 2 days. Some samples were incubated with rhodamine-labeled DBA lectin (10 µg/ml; Vector Laboratories, RL1032) for 12 h at 4°C. For fluorescent observation, the samples were counterstained with DAPI, cleared by CUBIC solution (Susaki et al., 2014) and observed under a fluorescence microscope (BX51N-34-FL2; Olympus) and stereomicroscope (SZX16 plus U-LH100HG; Olympus), as well as a TCS SP8 confocal laser microscope (Leica Microsystems).

### Morphometry and statistics

The ratio of SOX17- or SOX9-positive cells to total epithelial cells was estimated in the gallbladder body at 200× magnification using light microscopy. To quantify the density of PBG/PPG-like glandular structures in human gallbladder samples, their numbers per 100 µm were counted and estimated in the Alcian blue-stained sections prepared from the body segments. For mouse samples, whole-mount DBA-stained samples were photographed at 200× and the relative PBG/PPG number was estimated in each 100 µm section of the gallbladder and cystic duct regions.

Quantitative data are represented as mean±s.e.m. or number of individuals with a condition. Fisher's exact test was used to compare the proportions between groups. Statistically significant differences were identified using either two-sample *t*-tests or hypothesis testing for the difference in the population proportions. *P*<0.05 was considered statistically significant.

## Supplementary Material

Supplementary information
