# Supplementary material for: Gallbladder wall abnormality in biliary atresia of mouse Sox17+/− neonates and human infants
Source: Dis Model Mech. 2020 Apr 3;13(4):dmm042119. doi: 10.1242/dmm.042119 (PMC7132780; doi:10.1242/dmm.042119)
Supplement: Supplementary information [file dmm-13-042119-s1.pdf]

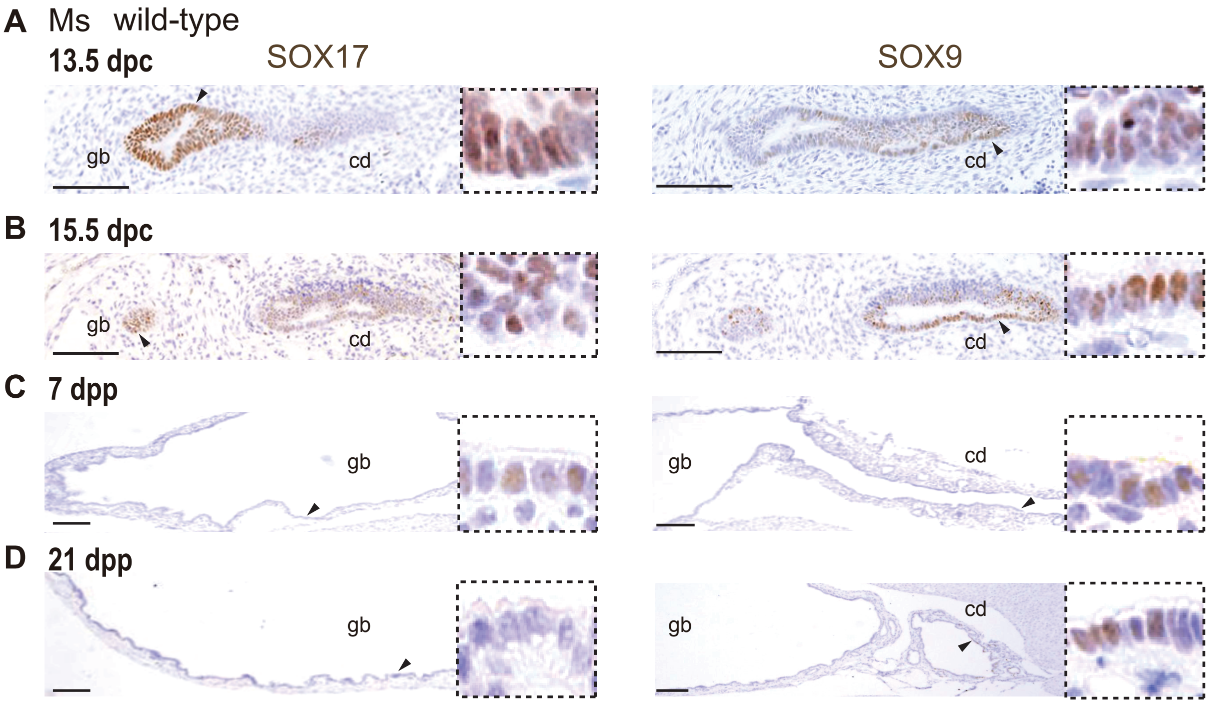

Fig. S1. SOX17 expression profiles in the gallbladder walls in fetal and postnatal development. A-D: Comparative anti-SOX17 (left) and anti-SOX9 (right) immunostaining of two serial sections of the gallbladder and cystic duct (sagittal plane) of the wild-type mouse embryos/pups at 13.5 day post-coitum (dpc) to 21 days post-partum (dpp). After the gallbladder and cystic duct tissues were dissected at each stage, the fixation, sectioning and staining were performed under the same condition. The insets show higher-magnification images of SOX17- or SOX9-positive epithelia in the gallbladder (left) or cystic duct (right) region, respectively. SOX17 expression levels in either cell density or signal intensity were high in the gallbladder wall at 13.5 dpc, and then reduced progressively with aging, reaching undetectable levels by 21 dpp (insets in “SOX17”). In contrast, there were no appreciable alterations in SOX9-positive cells in the cystic duct region (insets in “SOX9”). cd, cystic duct; gb, gallbladder; Ms, mouse. Scale bars: 100µm.

Disease Models & Mechanisms • Supplementary information
